# Correction: An allele-sharing, moment-based estimator of global, population-specific and population-pair *F*_ST_ under a general model of population structure

**DOI:** 10.1371/journal.pgen.1011133

**Published:** 2024-02-27

**Authors:** Jerome Goudet, Bruce S. Weir

In the Funding section, the grant number from the funder Swiss National Science Foundation for Research support is listed incorrectly. The correct grant number is: 31003A-179358.
