# Supplementary material for: An allele-sharing, moment-based estimator of global, population-specific and population-pair FST under a general model of population structure
Source: PLoS Genet. 2023 Nov 27;19(11):e1010871. doi: 10.1371/journal.pgen.1010871 (PMC10703327; doi:10.1371/journal.pgen.1010871)
Supplement: S1 Text — (PDF) [file pgen.1010871.s001.pdf]

S1 Text. A general reference point for kinship and F_{ST}

In general

$$F_{\mathrm{ST}}^{i \neq i'}(R) = \frac{\theta^{i \neq i'} - R}{1 - R}$$

where we take $R = \mathrm{mean}(\theta^{i \neq i'})$ and OS take $R = \min(\theta^{i \neq i'})$. For two different values R, R':

$$F_{\mathrm{ST}}^{i \neq i'}(R') = \frac{1 - R}{1 - R'} F_{\mathrm{ST}}^{i \neq i'}(R) + \frac{R - R'}{1 - R'}$$
