# Supplementary material for: An allele-sharing, moment-based estimator of global, population-specific and population-pair FST under a general model of population structure
Source: PLoS Genet. 2023 Nov 27;19(11):e1010871. doi: 10.1371/journal.pgen.1010871 (PMC10703327; doi:10.1371/journal.pgen.1010871)
Supplement: S2 Text — (PDF) [file pgen.1010871.s002.pdf]

S2 Text. Allele-sharing, kinship and inbreeding for a $k-$ploid species

for a $k-$ploid species, given a dosage matrix $\mathbf{X}$ with n rows (individuals) and L columns (loci) and with element $[j,l]$ corresponding to the number of copies of the designated allele for the jth individual at the lth locus, hence taking (integer) values between 0 and k, allele-sharing between the different possible dosages at a locus for that ploidy level are given in S1 Table. The allele-sharing matrix $\mathbf{A_S^k}$ is obtained by counting for each pair of individuals the proportion of loci in one of the k^2 states given in S1 Table and multiplying this proportion by the corresponding allele-sharing. This is efficiently done using the following:

$$\mathbf{A_S^k} = \frac{1}{2k^2L}(2\mathbf{X} - k\mathbf{J}_{[n,L]})(2\mathbf{X} - k\mathbf{J}_{[L,n]})^T + \frac{1}{2}\mathbf{J}_{[n,n]} \tag{1}$$

where $\mathbf{J}_{[a,b]}$ is a matrix of ones with a rows and b columns. An equivalent expression was independently derived by Bilton [1]

calling A_B the average of all the off-diagonal elements of $\mathbf{A_S^k}$, kinships are obtained as

$$\hat{\mathbf{K}}_{\mathbf{A_S}} = \frac{\mathbf{A_S^k} - \mathbf{J}A_B}{1 - A_B} \tag{2}$$

With the inbreeding coefficient for a $k-$ploid individual defined as the probability that two alleles drawn at random without replacement from the k the individual carries are identical (self kinship being the probability that two alleles drawn at random with replacement are identical), individual inbreeding coefficients are obtained from self kinship as:

$$\hat{F}_j^k = \frac{k \times \hat{K}_{AS_{jj}} - 1}{k - 1} \tag{3}$$

which reduces to the classical $\hat{F}_j = 2k_{AS_j} - 1$ for a diploid species ($\hat{F}_j^1$ is of course undefined for haploid species).

References

[1] Bilton, TP. Developing Statistical Methods for Genetic Analysis of Genotypes from Genotyping-By-Sequencing Data. University of Otago. 2020 Available from: `hdl.handle.net/10523/9975`.
