# Supplementary material for: An allele-sharing, moment-based estimator of global, population-specific and population-pair FST under a general model of population structure
Source: PLoS Genet. 2023 Nov 27;19(11):e1010871. doi: 10.1371/journal.pgen.1010871 (PMC10703327; doi:10.1371/journal.pgen.1010871)
Supplement: S3 Text — (PDF) [file pgen.1010871.s003.pdf]

S3 Text. Unequal sample sizes and subsampling populations

We illustrate using the river-system simulations from the main text the effect of unequal sample sizes from the different populations and of subsampling the populations.

We used the following sample sizes: $2, 4, 6, 8, 10, 20, 40$, each twice, and assigned these sample sizes at random to one of the 14 populations. We ran two replicates. The top row of S1 Fig shows the results. The different elements of $\hat{\mathbf{F}}_{\mathbf{ST}}$ are still well estimated, and RMSEs are of the same order of magnitude as those for equal sample sizes.

For the effect of subsampling populations, we drew seven populations out of the fourteen at random for three replicates and recalculated $\hat{\mathbf{F}}_{\mathbf{ST}}$. For this sampling scheme, expected values had also to be recalculated, since the reference set is not the fourteen populations but only the subset of seven. The results are illustrated on the bottom row of S1 Fig. The estimates are also unbiased.

The `R` code for these simulations is available from `https://github.com/jgx65/PlosGenetPopulationFST`
