# Supplementary material for: An allele-sharing, moment-based estimator of global, population-specific and population-pair FST under a general model of population structure
Source: PLoS Genet. 2023 Nov 27;19(11):e1010871. doi: 10.1371/journal.pgen.1010871 (PMC10703327; doi:10.1371/journal.pgen.1010871)
Supplement: S4 Text — (PDF) [file pgen.1010871.s004.pdf]

S4 Text. One thousand genomes population statistics

S2 Table shows continent-specific and S2 Fig shows population-specific estimates of F_{ST} obtained from phase 3 data of the 1000 genomes project for each of the 22 autosomes in the human genome.

S3 Fig shows RMSEs of $\hat{\mathbf{F}}_{\mathbf{ST}}$ for subsampling ten individuals per population, and using the complete dataset (2,504 individuals and all SNPs without filtering) as the expectation.

S3 Table gives estimates of $\hat{F}_{\mathrm{ST}}$ and $\hat{F}_{\mathrm{ST}}^{\mathrm{OS}}$ for each autosome. The pair of populations showing the minimum allele-sharing $\arg\min(\hat{\mathbf{F}}_{\mathrm{ST}})$ differs from one chromosome to the other. Mende from Sierra Leone (MSL) is always a member of the pair, but the other population varies. Because of this, $\hat{F}_{\mathrm{ST}}$ and $\hat{F}_{\mathrm{ST}}^{\mathrm{OS}}$ rank differently the 22 chromosomes (the correlation is 0.86 and the rank correlation is 0.82). Confidence intervals for $\hat{F}_{\mathrm{ST}}^{\mathrm{OS}}$ are larger than those for $\hat{F}_{\mathrm{ST}}$.

S4 Fig shows autosome estimates of $\hat{F}_{\mathrm{ST}}^{\mathrm{OS}}$ against $\hat{F}_{\mathrm{ST}}$ with their respective confidence intervals obtained by bootstrapping blocks of 100kb. CIs for $\hat{F}_{\mathrm{ST}}^{\mathrm{OS}}$ in blue are between 2.25 and 3.6 times wider than CIs for $\hat{F}_{\mathrm{ST}}$ in red.
