# Supplementary material for: An allele-sharing, moment-based estimator of global, population-specific and population-pair FST under a general model of population structure
Source: PLoS Genet. 2023 Nov 27;19(11):e1010871. doi: 10.1371/journal.pgen.1010871 (PMC10703327; doi:10.1371/journal.pgen.1010871)

S1 Fig. Effects of unequal sample sizes and subsampling populations on $\hat{\mathbf{F}}_{\mathbf{ST}}$. Sampling unequal numbers of individuals (top row) and subsampling populations (bottom row). Panel A, B: 2 replicates of using unequal sample sizes for estimating $\hat{\mathbf{F}}_{\mathbf{ST}}$. for each of the two replicates, we drew either $2, 4, 6, 8, 10, 20$ or 40 individuals (each sample size present twice) from each of the fourteen populations, assigning sample sizes at random to the fourteen populations; panel C: RMSEs for subsampling 50, 20, 10, 5, 2 individuals (same as panel B of Fig 5) and unequal sample sizes (var i); panels D-F: 3 replicates for the effect of subsampling 7 populations out of 14 from the river-system simulations. Elements of $\hat{\mathbf{F}}_{\mathbf{ST}}$ against their expectations

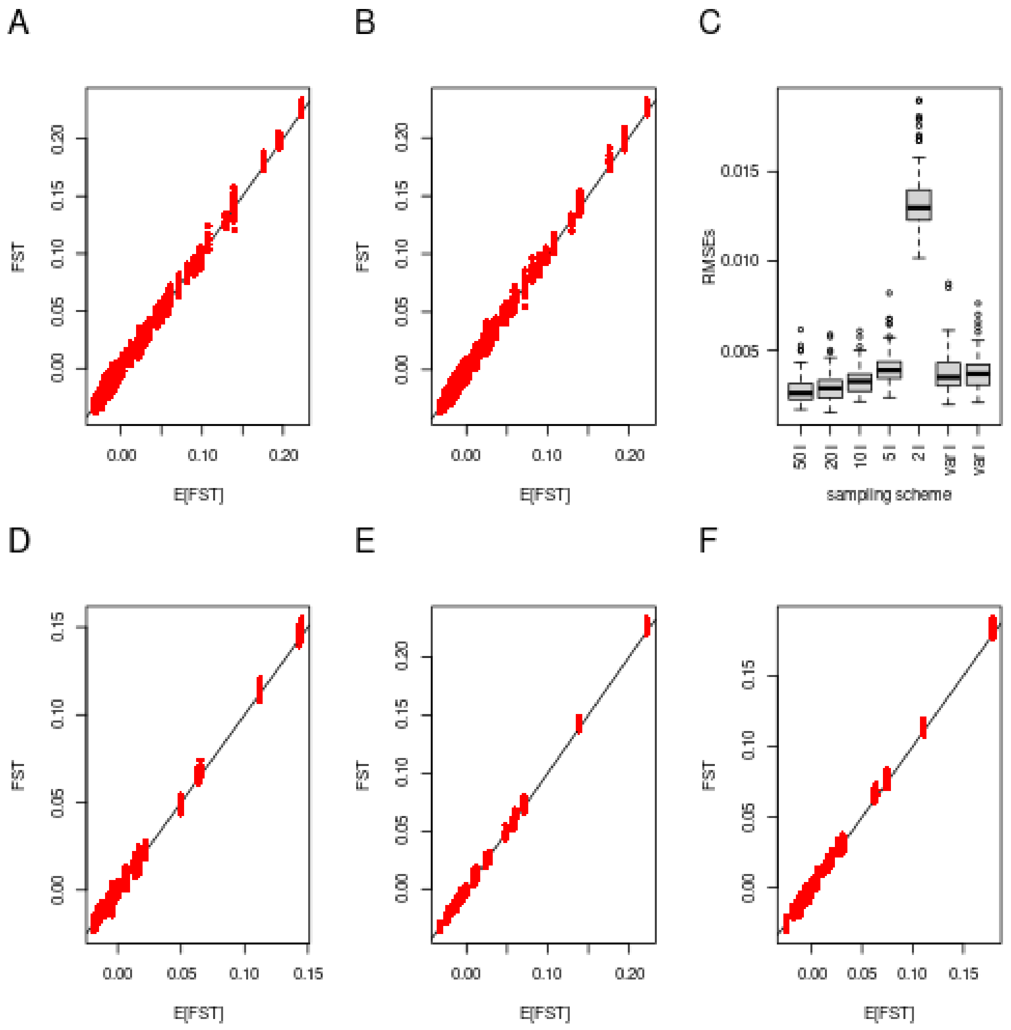

Supplement: S1 Fig — (PDF) [file pgen.1010871.s005.pdf]
