# Supplementary material for: An allele-sharing, moment-based estimator of global, population-specific and population-pair FST under a general model of population structure
Source: PLoS Genet. 2023 Nov 27;19(11):e1010871. doi: 10.1371/journal.pgen.1010871 (PMC10703327; doi:10.1371/journal.pgen.1010871)

S2 Fig. population-specific estimates of F_{ST} from the 1000 genomes Violin plots of the chromosome level, population-specific F_{ST} , using data from phase 3 of the 1000 genomes project. Populations are ordered by continent, Africa in gold, America in red, East Asia in green, Europe in blue and South Asia in purple. Average overall all populations is shown in grey. Each dot represents the estimate obtained from one of the 22 autosomes.

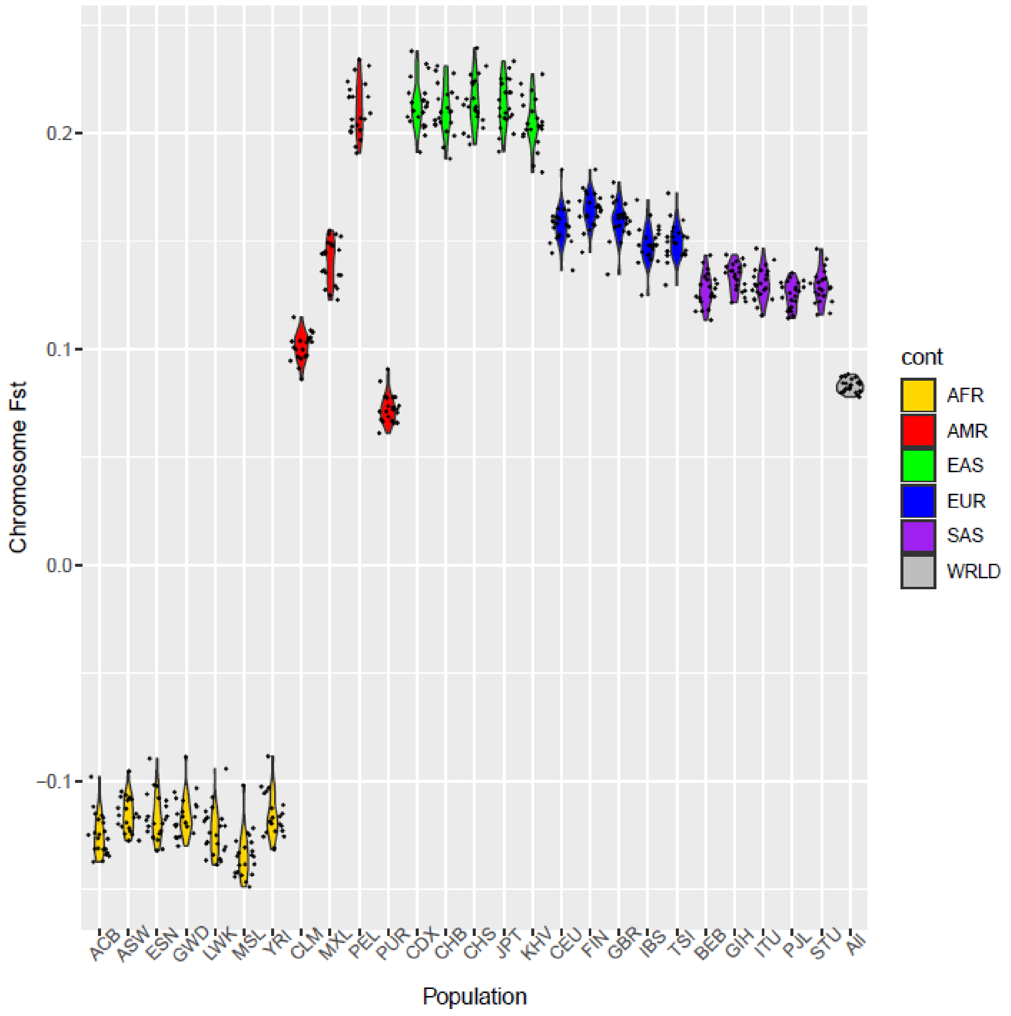

Supplement: S2 Fig — (PDF) [file pgen.1010871.s006.pdf]
