# Supplementary material for: An allele-sharing, moment-based estimator of global, population-specific and population-pair FST under a general model of population structure
Source: PLoS Genet. 2023 Nov 27;19(11):e1010871. doi: 10.1371/journal.pgen.1010871 (PMC10703327; doi:10.1371/journal.pgen.1010871)

S3 Fig. RMSEs of $\hat{\mathbf{F}}_{ST}$ in the 1000 genomes when subsampling 10 individuals per population. Color scale on the right hand side of the figure. The larger the size of the circle and the darker the color, the higher the RMSEs for this population/ population pair. All the populations that stand out are the admixed ones.

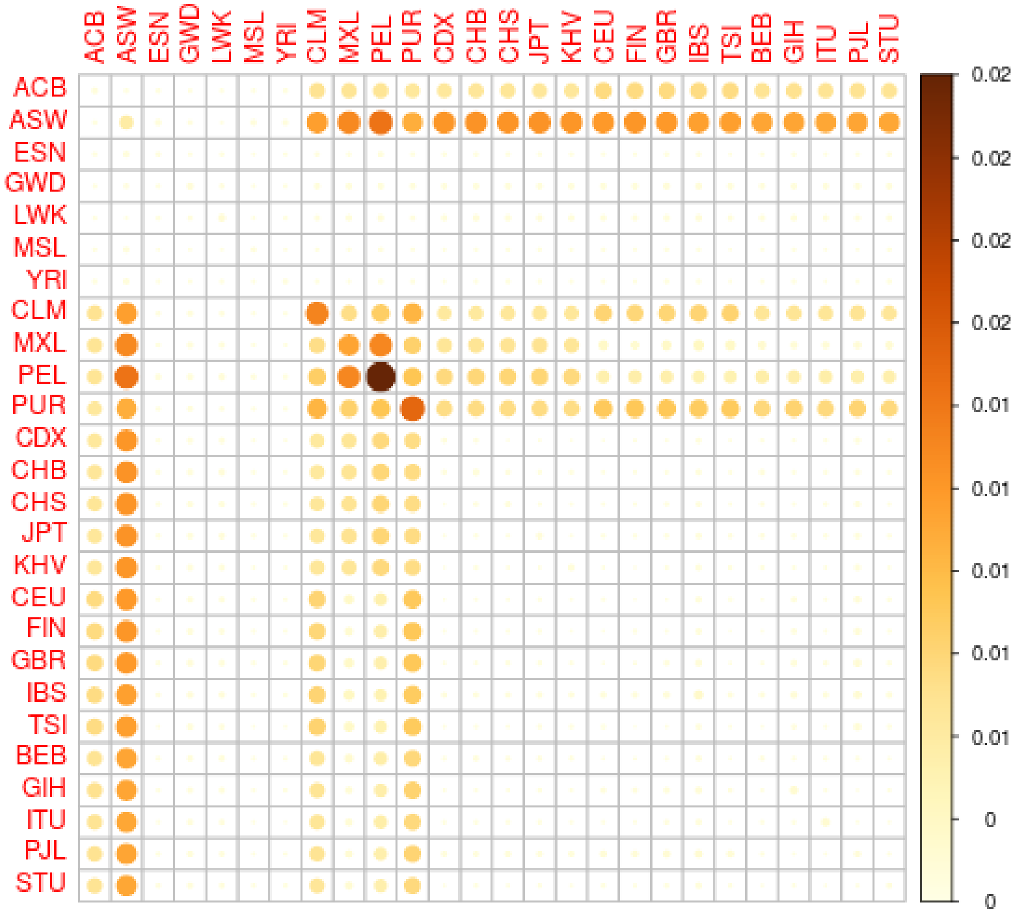

Supplement: S3 Fig — (PDF) [file pgen.1010871.s007.pdf]
