# Supplementary material for: An allele-sharing, moment-based estimator of global, population-specific and population-pair FST under a general model of population structure
Source: PLoS Genet. 2023 Nov 27;19(11):e1010871. doi: 10.1371/journal.pgen.1010871 (PMC10703327; doi:10.1371/journal.pgen.1010871)

S4 Fig. Chromosome specific $\hat{F}_{\mathrm{ST}}^{\mathrm{OS}}$ against $\hat{F}_{\mathrm{ST}}$ from the 1000 genomes data and their block bootstrap confidence intervals (CI). Note the x- and y-axis cover the same F_{ST} interval width (0.05). CIs for $\hat{F}_{\mathrm{ST}}^{\mathrm{OS}}$ in blue are between 2.25 and 3.6 times wider than CIs for $\hat{F}_{\mathrm{ST}}$ in red.

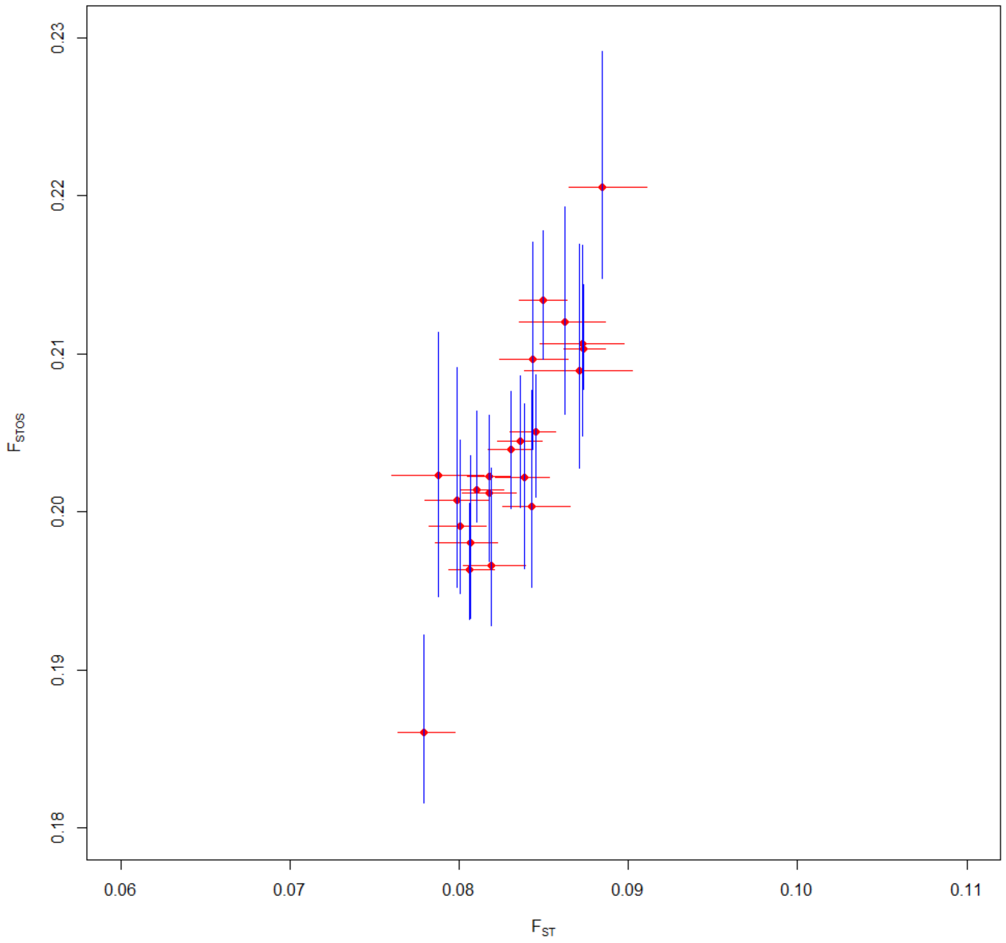

Supplement: S4 Fig — (PDF) [file pgen.1010871.s008.pdf]
