# Supplementary material for: An allele-sharing, moment-based estimator of global, population-specific and population-pair FST under a general model of population structure
Source: PLoS Genet. 2023 Nov 27;19(11):e1010871. doi: 10.1371/journal.pgen.1010871 (PMC10703327; doi:10.1371/journal.pgen.1010871)
Supplement: S1 Table — (PDF) [file pgen.1010871.s009.pdf]

S1 Table. Allele sharing probabilities $A_{jj'}$ between the different possible dosages for ploidies k from 1 to 6

$k=1$; j'		0	1
j	0	1.00	0.00
	1	0.00	1.00

$k=2$; j'		0	1	2
j	0	1.00	0.50	0.00
	1	0.50	0.50	0.50
	2	0.00	0.50	1.00

$k=3$; j'		0	1	2	3
j	0	1.00	0.67	0.33	0.00
	1	0.67	0.56	0.44	0.33
	2	0.33	0.44	0.56	0.67
	3	0.00	0.33	0.67	1.00

$k=4$; j'		0	1	2	3	4
j	0	1.00	0.75	0.50	0.25	0.00
	1	0.75	0.62	0.50	0.38	0.25
	2	0.50	0.50	0.50	0.50	0.50
	3	0.25	0.38	0.50	0.62	0.75
	4	0.00	0.25	0.50	0.75	1.00

$k=5$; j'		0	1	2	3	4	5
j	0	1.00	0.80	0.60	0.40	0.20	0.00
	1	0.80	0.68	0.56	0.44	0.32	0.20
	2	0.60	0.56	0.52	0.48	0.44	0.40
	3	0.40	0.44	0.48	0.52	0.56	0.60
	4	0.20	0.32	0.44	0.56	0.68	0.80
	5	0.00	0.20	0.40	0.60	0.80	1.00

$k=6$; j'		0	1	2	3	4	5	6
j	0	1.00	0.83	0.67	0.50	0.33	0.17	0.00
	1	0.83	0.72	0.61	0.50	0.39	0.28	0.17
	2	0.67	0.61	0.56	0.50	0.44	0.39	0.33
	3	0.50	0.50	0.50	0.50	0.50	0.50	0.50
	4	0.33	0.39	0.44	0.50	0.56	0.61	0.67
	5	0.17	0.28	0.39	0.50	0.61	0.72	0.83
	6	0.00	0.17	0.33	0.50	0.67	0.83	1.00
