# Supplementary material for: An allele-sharing, moment-based estimator of global, population-specific and population-pair FST under a general model of population structure
Source: PLoS Genet. 2023 Nov 27;19(11):e1010871. doi: 10.1371/journal.pgen.1010871 (PMC10703327; doi:10.1371/journal.pgen.1010871)
Supplement: S2 Table — (PDF) [file pgen.1010871.s010.pdf]

S2 Table. Estimates of chromosome and continent-specific F_{ST} from the 1000 genomes and their 95% confidence intervals obtained by block bootstrap, using 100kb blocks

Chrom.	AFR	AMR	EAS	EUR	SAS
1	-0.137	0.100	0.198	0.152	0.120
	(-0.145, -0.131)	(0.097, 0.103)	(0.193, 0.204)	(0.147, 0.157)	(0.116, 0.123)
2	-0.145	0.102	0.217	0.146	0.126
	(-0.151, -0.137)	(0.099, 0.106)	(0.211, 0.222)	(0.142, 0.151)	(0.122, 0.130)
3	-0.140	0.096	0.205	0.144	0.121
	(-0.147, -0.133)	(0.093, 0.099)	(0.198, 0.211)	(0.140, 0.149)	(0.118, 0.126)
4	-0.138	0.101	0.197	0.146	0.117
	(-0.145, -0.132)	(0.097, 0.105)	(0.192, 0.205)	(0.141, 0.150)	(0.113, 0.121)
5	-0.151	0.097	0.207	0.141	0.121
	(-0.157, -0.144)	(0.094, 0.100)	(0.202, 0.214)	(0.135, 0.145)	(0.116, 0.125)
6	-0.109	0.091	0.180	0.123	0.107
	(-0.118, -0.102)	(0.086, 0.094)	(0.172, 0.188)	(0.119, 0.130)	(0.103, 0.112)
7	-0.133	0.096	0.192	0.140	0.115
	(-0.142, -0.128)	(0.092, 0.099)	(0.186, 0.200)	(0.135, 0.144)	(0.111, 0.120)
8	-0.152	0.103	0.206	0.155	0.119
	(-0.161, -0.144)	(0.100, 0.107)	(0.199, 0.213)	(0.149, 0.161)	(0.116, 0.124)
9	-0.137	0.102	0.193	0.147	0.113
	(-0.146, -0.127)	(0.099, 0.107)	(0.184, 0.200)	(0.141, 0.153)	(0.107, 0.118)
10	-0.123	0.093	0.196	0.140	0.111
	(-0.132, -0.116)	(0.089, 0.097)	(0.188, 0.205)	(0.135, 0.148)	(0.107, 0.115)
11	-0.137	0.100	0.195	0.134	0.121
	(-0.145, -0.132)	(0.096, 0.103)	(0.188, 0.204)	(0.129, 0.140)	(0.116, 0.124)
12	-0.126	0.100	0.196	0.141	0.118
	(-0.134, -0.118)	(0.096, 0.103)	(0.188, 0.206)	(0.135, 0.146)	(0.113, 0.124)
13	-0.130	0.096	0.196	0.136	0.114
	(-0.138, -0.120)	(0.091, 0.101)	(0.185, 0.204)	(0.130, 0.142)	(0.108, 0.118)
14	-0.129	0.108	0.184	0.152	0.116
	(-0.137, -0.120)	(0.103, 0.113)	(0.176, 0.192)	(0.144, 0.159)	(0.111, 0.122)
15	-0.145	0.108	0.196	0.165	0.123
	(-0.157, -0.136)	(0.104, 0.114)	(0.190, 0.206)	(0.157, 0.173)	(0.116, 0.130)
16	-0.153	0.107	0.200	0.151	0.124
	(-0.163, -0.140)	(0.103, 0.112)	(0.191, 0.209)	(0.143, 0.157)	(0.118, 0.130)
17	-0.145	0.102	0.221	0.141	0.132
	(-0.155, -0.134)	(0.094, 0.108)	(0.211, 0.232)	(0.131, 0.151)	(0.125, 0.138)
18	-0.142	0.094	0.188	0.143	0.123
	(-0.155, -0.131)	(0.089, 0.099)	(0.179, 0.196)	(0.135, 0.150)	(0.117, 0.128)
19	-0.148	0.101	0.202	0.145	0.108
	(-0.160, -0.137)	(0.096, 0.106)	(0.191, 0.211)	(0.138, 0.154)	(0.103, 0.115)
20	-0.149	0.101	0.212	0.145	0.128
	(-0.161, -0.135)	(0.096, 0.107)	(0.203, 0.225)	(0.139, 0.152)	(0.122, 0.135)
21	-0.146	0.108	0.181	0.143	0.114
	(-0.161, -0.131)	(0.101, 0.115)	(0.172, 0.193)	(0.134, 0.153)	(0.104, 0.123)
22	-0.128	0.109	0.214	0.138	0.111
	(-0.142, -0.112)	(0.103, 0.115)	(0.197, 0.227)	(0.130, 0.145)	(0.102, 0.119)
