# Supplementary material for: An allele-sharing, moment-based estimator of global, population-specific and population-pair FST under a general model of population structure
Source: PLoS Genet. 2023 Nov 27;19(11):e1010871. doi: 10.1371/journal.pgen.1010871 (PMC10703327; doi:10.1371/journal.pgen.1010871)
Supplement: S3 Table — (PDF) [file pgen.1010871.s011.pdf]

S3 Table. Comparison of $\hat{F}_{\mathrm{ST}}$ (the average of population-specific $\hat{F}^{i}_{\mathrm{ST}}$s, Eq 8) and $\hat{F}^{\mathrm{OS}}_{\mathrm{ST}}$ in the 1000 Genomes data set for each chromosome. Confidence intervals obtained by block bootstrap, using 100kb blocks

Chrom	$\arg\min(\hat{\mathbf{F}}_{\mathrm{ST}})$	$\min(\hat{\mathbf{F}}_{\mathrm{ST}})$	$\hat{F}_{\mathrm{ST}}$	$\hat{F}^{\mathrm{OS}}_{\mathrm{ST}}$
1	MSL-PEL	-0.152	0.085	0.205
			(0.083, 0.086)	(0.201, 0.209)
2	MSL-KHV	-0.156	0.087	0.210
			(0.086, 0.089)	(0.208, 0.214)
3	MSL-CHB	-0.152	0.084	0.204
			(0.082, 0.085)	(0.200, 0.209)
4	MSL-MXL	-0.152	0.083	0.204
			(0.082, 0.084)	(0.200, 0.208)
5	MSL-ITU	-0.151	0.082	0.202
			(0.080, 0.083)	(0.199, 0.206)
6	MSL-JPT	-0.133	0.078	0.186
			(0.076, 0.080)	(0.182, 0.192)
7	MSL-CDX	-0.144	0.081	0.196
			(0.079, 0.082)	(0.193, 0.201)
8	MSL-PEL	-0.163	0.085	0.213
			(0.084, 0.086)	(0.210, 0.218)
9	MSL-PJL	-0.149	0.082	0.201
			(0.080, 0.083)	(0.197, 0.206)
10	MSL-ITU	-0.143	0.082	0.197
			(0.080, 0.084)	(0.193, 0.203)
11	MSL-CEU	-0.151	0.081	0.201
			(0.080, 0.083)	(0.199, 0.206)
12	MSL-CEU	-0.148	0.084	0.202
			(0.082, 0.085)	(0.196, 0.207)
13	MSL-JPT	-0.146	0.081	0.198
			(0.079, 0.082)	(0.193, 0.204)
14	MSL-CEU	-0.145	0.084	0.200
			(0.083, 0.087)	(0.195, 0.208)
15	MSL-PEL	-0.156	0.087	0.211
			(0.085, 0.090)	(0.205, 0.217)
16	MSL-PEL	-0.159	0.084	0.210
			(0.082, 0.086)	(0.204, 0.217)
17	MSL-TSI	-0.169	0.088	0.221
			(0.086, 0.091)	(0.215, 0.229)
18	MSL-CEU	-0.149	0.080	0.199
			(0.078, 0.082)	(0.195, 0.205)
19	MSL-GIH	-0.151	0.080	0.201
			(0.078, 0.082)	(0.195, 0.209)
20	MSL-CDX	-0.160	0.086	0.212
			(0.084, 0.089)	(0.206, 0.219)
21	MSL-KHV	-0.155	0.079	0.202
			(0.076, 0.081)	(0.195, 0.211)
22	MSL-PEL	-0.154	0.087	0.209
			(0.084, 0.090)	(0.203, 0.217)
All	MSL-PEL	-0.149	0.083	0.202
