# Supplementary material for: An allele-sharing, moment-based estimator of global, population-specific and population-pair FST under a general model of population structure
Source: PLoS Genet. 2023 Nov 27;19(11):e1010871. doi: 10.1371/journal.pgen.1010871 (PMC10703327; doi:10.1371/journal.pgen.1010871)
Supplement: Attachment — Submitted filename: Answers_to_reviewers.pdf [file pgen.1010871.s012.pdf]

Dear Editor,

Please find enclosed a revised version of our manuscript “An allele-sharing, moment-based estimator of global, population-specific and population-pair FST under a general model of population structure.” following the editors and reviewers comments.

We are extremely grateful to the editors and the reviewers, as these comments were extremely helpful and thoughtful. We believe they helped us clarify the manuscript.

Below, we give a point by point answer to all comments (in red), and the corresponding line numbers for changes in the manuscript.

We look forward to hearing whether the manuscript is suitable for publication in Plos Genetics.

Best wishes

Jérôme Goudet & Bruce Weir

Dear Dr Goudet,

Thank you very much for submitting your Methods entitled 'An allele-sharing, moment-based estimator of global, population specific and population pairs FST under a general model of population structure.' to PLOS Genetics.

The manuscript was fully evaluated at the editorial level and by independent peer reviewers. The reviewers appreciated the attention to an important topic but identified some concerns that we ask you address in a revised manuscript. Particular attention should be paid to the following issues:

As noted by reviewer 1 the role of the Ochoa & Storey (O&S) estimator in the development of the ms is not clear. Generally speaking, Fst's are comparisons of some probabilities of identity relative to some others, and a relevant case in practice is that the "others" are defined from the current, actually observed populations (as in this ms). It appears that O&S's claim that previous estimators are biased rests on applying the general concept in a different way, according to which some ancestral population is considered as a reference. One can debate about the definition of the parameters, as well as about the fairness of assessing bias of estimators relative to a parameter they were not conceived to estimate. The ms restates correctly the definition of the parameters estimated by various estimators, but it also appears to proceed as if the parameter to be estimated should be the one that is easiest to estimate. To

address the comments of reviewer 1, the authors should perhaps consider that if the O&S parameter were really the one to estimate, then the results of the ms with respect to estimators would not be so important, and therefore discuss O&S's definition.

We now describe in the introduction and definition of FST section O&S work and how it links with ours. We also explain why this paper relates to our work (L 28-31, 96-98, 134-140)

In addition, there are some ambiguities about what exactly is new in this ms. In particular, the introductory review of the literature does not highlight the distinctive features of the estimator discussed here. The estimator uses a general idea of allele sharing and appears to avoid any form of weighting according to per-population sample size. This is distinctly relevant when populations have different expected genetic diversities, so it make sense to apply it to a general migration model (not assuming identical population sizes or migration rates) as done in this ms. It is this combination of features that is new.

We are thankful to the editor for pointing this out. We now clarify this point, in particular the difference with Weir & Hill (L 80-82, 454-457)

The "general population model" is not new per se. Yet, reading the second reviewer's comment, it looks as if it is. This suggests that although various references are cited in the ms after its formulation, the ms could perhaps be more explicit from the start about earlier formulations of the model.

We've added a reference to the work of Nagylaki (1982) and we also explain the specific feature of the model and our estimators L 147, 170-183

As noted by reviewer 2 the sample sizes in the simulations are not clear, although this is an important consideration in the definition of the estimator (the allele-sharing estimator yields the same estimates as alternative, widely used estimators such as the one of Weir and Cockerham 1984, when the sample sizes are the same among populations).

This is a very good point. It is unusual to adopt sampling strategies with an imbalance in the samples, but here, and in order to distinguish the Weir & Goudet estimator from Weir and Cockerham, it makes full sense. We've added text in the results section and a section "Unequal sample sizes and subsampling populations" in the supporting information where we illustrate the consequences of unequal sample sizes and subsampling only a portion of the populations on elements of the estimator of the FST matrix.

We therefore ask you to modify the manuscript according to the review recommendations. Your revisions should address the specific points made by each reviewer.

In addition we ask that you:
1) Provide a detailed list of your responses to the review comments and a description of the changes you have made in the manuscript.
2) Upload a Striking Image with a corresponding caption to accompany your manuscript if one is available (either a new image or an existing one from within your manuscript). If this image is judged to be suitable, it may be featured on our website. Images should ideally be

high resolution, eye-catching, single panel square images. For examples, please browse our archive. If your image is from someone other than yourself, please ensure that the artist has read and agreed to the terms and conditions of the Creative Commons Attribution License. Note: we cannot publish copyrighted images.

We hope to receive your revised manuscript within the next 30 days. If you anticipate any delay in its return, we would ask you to let us know the expected resubmission date by email to plosgenetics@plos.org.

If present, accompanying reviewer attachments should be included with this email; please notify the journal office if any appear to be missing. They will also be available for download from the link below. You can use this link to log into the system when you are ready to submit a revised version, having first consulted our Submission Checklist.

While revising your submission, please upload your figure files to the Preflight Analysis and Conversion Engine (PACE) digital diagnostic tool. PACE helps ensure that figures meet PLOS requirements. To use PACE, you must first register as a user. Then, login and navigate to the UPLOAD tab, where you will find detailed instructions on how to use the tool. If you encounter any issues or have any questions when using PACE, please email us at figures@plos.org.

Please be aware that our data availability policy requires that all numerical data underlying graphs or summary statistics are included with the submission, and you will need to provide this upon resubmission if not already present. In addition, we do not permit the inclusion of phrases such as “data not shown” or “unpublished results” in manuscripts. All points should be backed up by data provided with the submission.

To enhance the reproducibility of your results, we recommend that you deposit your laboratory protocols in protocols.io, where a protocol can be assigned its own identifier (DOI) such that it can be cited independently in the future. Additionally, PLOS ONE offers an option to publish peer-reviewed clinical study protocols. Read more information on sharing protocols at https://plos.org/protocols?utm_medium=editorial-email&utm_source=authorletters&utm_campaign=protocols

Please review your reference list to ensure that it is complete and correct. If you have cited papers that have been retracted, please include the rationale for doing so in the manuscript text, or remove these references and replace them with relevant current references. Any changes to the reference list should be mentioned in the rebuttal letter that accompanies your revised manuscript. If you need to cite a retracted article, indicate the article’s retracted status in the References list and also include a citation and full reference for the retraction notice.

PLOS has incorporated Similarity Check, powered by iThenticate, into its journal-wide submission system in order to screen submitted content for originality before publication. Each PLOS journal undertakes screening on a proportion of submitted articles. You will be contacted if needed following the screening process.

To resubmit, you will need to go to the link below and ‘Revise Submission’ in the ‘Submissions Needing Revision’ folder.

Please let us know if you have any questions while making these revisions.

Yours sincerely,

François Rousset
Guest Editor
PLOS Genetics

David Balding
Section Editor
PLOS Genetics

Additional Guest Editor's comments

Miscellaneous:

l.137: I would avoid hasty claims about generality of the equation here as they overlook important issues. For example, with random local extinctions, the form of the recursion may still be correct conditionally on realized extinctions, but if the past history of extinctions is not known, the expected identities and Fst values that may be of interest in any inferential context are expectations over a distribution of past extinction events, and the conditional recursion is not sufficient to compute these expectations.

We agree with the editor and we removed this sentence

Mutation is a parameter of model but seems to disappear afterwards. Was it set to zero in Fig. 1, for example?

The mutation rate is 10^-8 for all three models. We now specify this in the caption of the figure. We also renamed the 9 panels (A-H) and give details about migration rate and population sizes in the figure caption.

p.6 l.18-19: I wonder whether the authors really meant to write "correlation" rather than coancestry in this sentence about correlation ... relative to ... correlation. Speaking about correlation here seems to leave something undefined in the reader's mind (namely, correlation of what relative to what?).

changed to correlation of allelic indicators L 19

Reviewer's **Comments to the Authors:**

Reviewer #1: The authors describe IBD probabilities between all pair of populations under a general model of population structure. They use these IBD probabilities to define matrix-FST, based on the classic definition of F statistics by Rousset (2004). In this matrix-FST, diagonal elements correspond to what it is known as population-specific FST in the literature, and their average correspond to FST. Off-diagonal elements are a somehow new quantity and their

interest in conservation genetics is discussed by the authors. Then, the authors present an allele-sharing based estimator of this matrix-FST and they study its statistical properties and compare it to a Bayesian estimator of FST. In addition, the authors discuss another recently proposed estimator of FST based on a different definition of F statistics (Ochoa & Storey, 2022).

I found their work interesting both from a theoretical point of view with their general model for FST, which is a key parameter in population genetics, and from a practical point of view, since computationally efficient estimators are useful with the ever increasing datasets in population genetics. The research is conducted rigorously and conclusions are supported by results. I have not scientific concerns about the originality and validity of this work. However, I think the text needs a thorough revision to improve clarity. The logical connection between different parts of the article needs to be better established. I recommend the text to be revised before it is accepted for publication.

we've tried to make links between sections smoother and adding text to this end in several places in the manuscript

Main concern:

1) The current version of the manuscript does not clearly present the relevance of the analysis of the work of Ochoa & Storey (2022) on FST. In the methods section Ochoa & Storey FST is treated in a brief and separate subsection and results are presented only in the appendix. However, almost half of the discussion is dedicated to it. My impression is that it is not explained to the reader why this particular work is important in the context of their general model and estimator. The text, possibly in the introduction section, needs to be more explicit on what is the role of the Ochoa & Storey FST on the conception, development and results of the rest of work presented.

Ochoa & Storey claimed the Weir & Goudet estimator of FST is biased (see for instance their fig 4). Here we show it is not the case. We've added sentences explaining this at the end of the introduction and in several other places L 96-98, 134-141, 612-619

Minor comments:

2) The section "Statistical properties of FST" (pages 10-11) presents the evaluation of the estimators in a confusing way, in my opinion. I think a general revision would improve the clarity. In particular, I suggest to describe simulations, even if it is briefly (e.g. "a coalescent-with-recombination simulation…", "simulation of allele frequencies of independent loci…" or other relevant description), before specifying the software used for them.

We have tried to clarify this section and followed the reviewer's advice. L 330-337, 365-366

3) The results section feels like an extended figure caption. I think the reader will follow better the text if the each paragraph starts with a sentence that announces the content of that

paragraph rather than the content of a figure. “In the finite island model… (Figure 3)” instead of “Figure 3 shows the results…”.

We have rephrased the paragraph following the reviewer advice. L 396, 414, 428

4) Expected FST in different figures are represented with circles of different colors. These circles are also of different size, and the size seems to be correlated with the absolute FST value. However this is not specified. Please clarify in the caption of the figure to avoid confusion.

We have now clarified in the figure captions that both color and size of the circle describe how large the element of the matrix is.

5) Use standard conventions for notation: italic letters for variables, including vectors and matrices, and roman letters for labels. In FST, F should be italic (and bold for matrix-FST) and ST in roman. In any case, use an uniform notation, matrix-FST is presented sometimes in italics and sometimes in roman.

We’ve checked through the document and replaced labels (ST and OS) with Roman letters

6) Paragraph in lines 510-517 uses subindex “W” for FST. If I understood correctly, this subindex needs to be removed to use the same notation that the rest of the article. However, if this has a specific meaning to differentiate to other FST, it needs to be clearly defined in the text.

This W was a left over and we have now removed the W in this section

7) Write acronyms in capital letters for better readability (e.g. IBD instead of ibd).

done

8) Label panel in figures for better reference.

done

9) Author summary is missing.

We have now written an Author sumary

10) Line 117 word “from” duplicated.

done

Reviewer #2: I have read J. Goudet's and B. Weir's manuscript with an immense interest and I must confess, quite an admiration. I have unfortunately not been able to take enough time to review the mathematical aspects of the proposed estimator so my apologies for any lack of comments on this aspect.

After a brief review of FST, including the population-specific definition and their estimators, the authors provide the general expectations for the recurrence of coancestry values between any pair of individuals provided a matrix of migration, thus allowing the calculation for an arbitrary population model. They then propose a moment-estimator for FST and the population-pair-specific FST_P based on the allele sharing between pairs of individuals. Using simulations in population models of increasing complexity, including a river system with two tributaries, they investigate the performance of their estimators and its sensitivity to variable SNP sampling and sample sizes. Lastly, they apply their estimator to the human 1KG dataset and compare the expectations to the ones of another moment-based estimator using a different scaling for the reference population.

I have found the manuscript of excellent quality. The performance analyses are convincing and the discussion thorough and thoughtful. The authors' estimator has a much higher accuracy than Bayesian estimates in all situations. Its performance is also impressive even for low data quality. I have only very minor comments, and a general call for making the most technical parts slightly more accessible. Congratulations for this work, and looking forward to using this new estimator. Also, I wanted to stress that the relevance of this estimator for conservation genomics, as explained in the Discussion, is of immense interest, since it can quickly provide a set of hypotheses for further modeling analyses. This can be very useful for data exploration and could as well serve as an unbiased summary statistics for ABC studies?

We thank the reviewer for this very positive comment, and we agree the proposed model could be very useful for data exploration, this is certainly an area where we intend to investigate further.

___Things I would be interested to know more about

- One of the advantage of the estimator is its performance even for low sampling size and SNP density. How would it perform for aDNA-style data, including pseudodiploidy? Would it be possible to report a RMSE in such case? I think this would be very useful to show the usability of this method for ancient DNA research.

We thank the reviewer for pointing this out, and we've added a sentence in this discussion pointing to the issue of low-coverage sequencing /pool seq / aDNA as something needing further scrutiny L 511-513

- Ochoa & Storey's F_ST^OS estimator uses the minimum AS as a reference point, instead of your estimator using the average. For me, the minimum would lead to a very strong sensitivity to the population sampling design. For instance, suffice that one misses the global population minimum by picking a local one, this would bias the estimate. Instead, what would the results be if using a statistics like the percentile at 10%? A bit on the same topic, you use the average

between population, but this could equally be sensitive to the sampling design and to population outliers. Would it be possible to give an intuition, or even, a simulation case using a heterogeneous sampling design with a more robust quantity (e.g. median)?

We've added a paragraph in the discussion L 575-588

- Regarding the sampling design, I may have missed something, but it seems that you consider nearly all the populations in your simulated examples? If this is so indeed, could you perform one small performance study in which you consider only a subset of the populations, with a uniform sampling and with a heterogeneous one? e.g. a bit akin to what we have with the 1KG.

Using a subset of the populations changes the reference set and therefore changes the scaling of the estimates but not their ranking. We've added a section "Unequal sample sizes and subsampling populations" in supporting information illustrating this point.

- I did not clearly understand why the sensitivity to sampling size would be higher for admixed populations (e.g. Puerto Ricans in the 1KG)?

Fig S2 shows the RMSEs when subsampling individuals are largest for elements of the FST matrix where one or both members are from an admixed population (ACB, ASW, CLM, MXL, PEL and PUR). This is because the subsamples will contain (by chance) a different proportion of individuals from each ancestry. we've added words to explain this L 474-476

___For clarification (or confirmation)

- I got a bit confused by the RMSE calculation across the simulation and the application (1KG) cases: what is the reference value that you compare with?

Changed, we used the full data set for expectation (i.e. the ~78 M SNPs for the 2504 individuals)

___General

- In general, figures lack resolutions and some were hard to read (labels). Also, the numbers of the scale overlap the color scale, making the "-" sign invisible. On the `FST vs. E[FST]` plots, would be nice to report the CIs.

We've redrawn the figures, added labels for panels and redrawn the color scale. Adding CIs for the 100 (island and stepping stone models) or 196 (river system) matrix elements would make the graph extremely busy and we feel showing the point estimates gives a better idea of the variation.

- I found the description of the methods lacking a bit details, for instance it would be nice to have the commands (if not a full script) for the simulations, including with msprime, as well as for the analyses using fs.dosage().

The code is now available from the following github site:

https://github.com/jgx65/PlosGenetPopulationFST

- In addition, I am lacking info about the methodological analysis of the 1KG dataset. Was there any filtering applied to the SNP set? Was the raw data directly used? If so, or if not, please clarify.

No, no filtering, the raw data were directly used, this is now clearly specified.

___Specific comments

- L33 A quick conceptual definition of what population-specific FST measures would be nice

We've added a sentence in the introduction, L 40-42.

- L49 The "F_ST's" notation is a bit confusing? "'s" is for plural? I am not sure it is necessary.

We removed the trailing s

- L111 Can you be more specific, what "migration rates" do you refer to in this case? I understood that the migration rates you consider afterwards are the intrinsic probabilities.

We have defined the elements of the migration matrix as "elements of the matrix $m_{ii'}$ are the proportions of alleles in row population i that are from column population i' in the previous generation, including the case of $i = i'$. The only constraint on M is that all its elements are positive or zero, and each row sums to 1".

We are not sure how we could be more specific?

- L117 Remove one "from"

Done

- L122 Clarify the direction of time: forward or backward, also clarify for the simulators

done

- Eq2 Would be nice to conceptually decompose the equation

We have written words to explain the meaning of the two terms

- Fig1 Correct: "sends", "receives" | Change colors of the lines according to N | Why m in stepping-stone is 1e-2 and not 1e-3 like the others? | Define the x-axis (possibly in legend): generations from what? | Why showing the absolute coancestry and not the actual value?

We've corrected the typos, and changed the colours according to effective sizes. We chose the migration rate to illustrate best the dynamics of coancestries and FST, and a migration rate of 10^ -2 illustrated better the stepping stone. Generation 0 is before the populations split, and hence has a coancestry of 0. We removed the "absolute" qualifier to coancestry, as it was indeed confusing.

- L212 Define “r”

Done (r is the number of populations)

- L286 Correct: “are entered”

Corrected

- L336: What is “c” here? Between the ends of the chromosome? The unit should be number of crossing-overs.

We’ve modified the text specifying now that recombination between adjacent base pairs is 10^-8, and hence 1 crossing per per chromosome is expected on average.

- L342 Correct: “expected”

Corrected

- L398 I would just say “more heterozygous”, I do not know up to what % you consider it to be highly. The difference is significant but not extreme with some other non-African pops.

corrected

- L412 Sorry I may have missed it, but how were loci subsampled? Uniform according to physical position?

Yes, we’ve added this in the material and methods section when we described estimation of RMSE for the 1000 genomes

- Fig 6 Report in the caption the total number of SNPs from the original dataset (what provides the reference value)

Corrected

- L462-468 The logic of the paragraph is not crystal-clear, could you develop it or clarify it?

We’ve rephrased this paragraph we hope it is now clearer as this is an important and novel point L 531-545

- L470 What do you mean by “the size”? Level?

We’ve reworded the sentence. By size, we mean how large it is (L 546)

___Code

I tested the code (hierfstat) package and could run the functions smoothly to estimate FST using simulated individuals. Documentation is present and complete.

Reviewer #3: The paper provides a well-written and constructed overview of recent papers based on FST, and provides some useful results on the accuracy of their moment-based estimator, first described in Weir and Goudet (2017). The authors compare and contrast their approach with two recent methods described in PLoS Genetics. With three such recent papers in PLoS Genetics, FST is clearly well-served! I just have some relatively minor points:

Eqn 2. The need for \mu in this derivation. It's probably not the best place to put it, but somewhere in the ms, a strength of which is its review of previous work on FST, I would like to see some mention of Slatkin's 1991 definition of FST (at least, as reformulated in Rousset's 1996 Genetics paper, defined in terms of (t_b - t_w)/t_b rather than the GST-way that Slatkin used [no need to mention msats!]). The advantage of Slatkin's definition is that it is then purely genealogical, and could e.g. be applied as a predictive quantity from some coalescent/likelihood/Bayesian algorithm to infer demographic history. Otherwise one tends to have to brush the mutation rate under the carpet. Maybe it is OK for individual SNPs, but for e.g. haplotype-based FSTs one then needs to special-plead the different values you get for different marker types.

We have now added in the section definition of FST a reference to the work of Slatkin (1991) and Rousset (1996) L 118-123

333-338. I'm a little unclear on this description. So this is a copy of what they did with sim.genot.metapop.t but with msprime? So presumably something needs to be said about mutation rate as well as map length? Also '20 chromosomes' refers to sample size?

Agreed, we now give more details and provide the code we used for both simulation methods L387-393

Results: The AFM method clearly does not work very well. Yet the authors do little to unpick what the problem is. Maybe, as the authors note, the computational demands of the method make it not worth it. On the other hand, maybe some discussion of this would be helpful epistemologically. Some lines of thought... Maybe the admixture model assumed in AFM is not very good? The authors note that Gaggiotti and Foll showed satisfactory performance of their likelihood method for the island model, so perhaps their method could be applied in addition to AFM for the island case (Fig 3) to see whether it is an issue intrinsic to the model. To some extent, there is always a bit of an issue to working out the optimal point estimator from a likelihood-based method because the mle is often biased. Maybe try to separate this out in the RMSE? It looks as though RAFM uses MCMC, so is the run-length/convergence an issue?

We agree we did not pay enough attention to explain the behaviour of the Bayesian estimator. Indeed, the computational demand makes it impractical for genome size data set and we now specify this more clearly. We have also redrawn the lower panels of Fig 3, 4 and 5 where we compare the moment and the Bayesian estimator with the same number of loci, keeping the same scale for the y axes. This helped show the Bayesian estimator is less variable, but biased, in particular for large positive and negative elements of the matrix. It seems as if there is a strong prior to remaining at 0, even in the island model. We've added words to explain this L 443-446

Gaggiotti and Foll method provides estimates for the diagonal elements of the FST matrix only, not for the whole matrix, we thus feel comparing their methods to ours does not belong to this manuscript (and may be redundant with the simulation work already done by Gaggiotti and Foll (2010)). L 349-343

Have all data underlying the figures and results presented in the manuscript been provided?
Large-scale datasets should be made available via a public repository as described in the *PLOS Genetics* data availability policy, and numerical data that underlies graphs or summary statistics should be provided in spreadsheet form as supporting information.

Reviewer #1: **No:** Current version of the manuscript does not seem to comply with the standards required by PLoS Genetics. Simulated data generated to evaluate the method that represent the main body of results of the article is not made available. The authors should provide the code to generate the simulated data used in this work.

The code for the simulations and figure can be found at

https://github.com/jgx65/PlosGenetPopulationFST

We've added this URL at the end of the method section

Reviewer #2: Yes

Reviewer #3: Yes

PLOS authors have the option to publish the peer review history of their article (what does this mean?). If published, this will include your full peer review and any attached files.

If you choose "no", your identity will remain anonymous but your review may still be made public.

Do you want your identity to be public for this peer review? For information about this choice, including consent withdrawal, please see our Privacy Policy.

Reviewer #1: **Yes:** Miguel de Navascués

Reviewer #2: No

Reviewer #3: No
